# The effect of hospital safety climate on nurses' turnover intention: the mediating role of work engagement

**DOI:** 10.3389/fpubh.2026.1789098

**Published:** 2026-05-04

**Authors:** Niu Yang, Jiehao Zhuang, Xuanhao Fan, Ziyi Xiong, Zhongqing Chen, Tiemei Shen, Xiaoxia Wang, Chunyan Sun, Guanrong Zhang, Lifang Chen, Huigen Huang

**Affiliations:** 1 Department of Nursing, Jinan University, Guangzhou, Guangdong, China; 2 Department of Nursing, Guangdong Provincial People's Hospital (Guangdong Academy of Medical Sciences), Southern Medical University, Guangzhou, Guangdong, China; 3 Department of Nursing, Guangdong Pharmaceutical University, Guangzhou, Guangdong, China; 4 Information Management Department, Guangdong Provincial People's Hospital (Guangdong Academy of Medical Sciences), Southern Medical University, Guangzhou, Guangdong, China; 5 Department of Nursing, Zhujiang Hospital, Southern Medical University, Guangzhou, Guangdong, China

**Keywords:** hospital safety climate, mediating effect, nurses, turnover intention, work engagement

## Abstract

**Background:**

Nurses are vital in addressing global healthcare workforce shortages. The shortage of nurses and high turnover rates are concerned with the quality of care. To further explore the mechanisms underlying nurse turnover, this study focuses on the mediating role of work engagement, examining the relationship between hospital safety climate, work engagement, and turnover intention.

**Methods:**

This was a cross-sectional research design of nurses in hospitals in Guangdong Province using a convenience sampling method. General information survey, hospital safety climate scale, work engagement scale, and turnover intention scale were adopted. The obtained data were analyzed through descriptive statistics, Pearson's correlation coefficient, and the mediating effect of work engagement was tested through the PROCESS macro mediation model in SPSS.

**Results:**

The hospital safety climate was positively correlated with work engagement (*r* = 0.549, *P* < 0.01), and negatively correlated with turnover intention (*r* = −0.324, *P* < 0.01). Work engagement was negatively correlated with turnover intention (*r* = −0.400, *P* < 0.01). The mediating effect of work engagement on the relationship between hospital safety climate and turnover intention has been demonstrated. The mediating effect is significant, with a value of−0.175, representing 53.77% of the total effect.

**Conclusions:**

The turnover intention of nurses in Guangdong Province is high. Work engagement plays a partial mediating role between hospital safety climate and turnover intention among nurses. This study further enriches JD-R theory and new ideas for nurse managers to take measures to stabilize the nursing workforce and reduce turnover.

## 1 Introduction

Nurses are the largest healthcare workforce in the world and play a critical role in the delivery of healthcare services. With rapid socioeconomic development and shifts in population structure, nursing shortages have become a global issue (1). The nursing workforce crisis has become the greatest threat to global health (2). According to the World Health Organization (WHO) report, it was estimated that there will be a shortage of 7.2 million health workers to deliver healthcare services worldwide, and by 2035, the demand for nursing will reach 12.9 million (3). In a study by the National Council of State Boards of Nursing (NCSBN), 100,000 nurses left the workforce during the pandemic in the USA and by 2027, almost one-fifth of 4.5 million total registered nurses will intend to leave the workforce (4). The shortage of nurses in China is a long-standing problem in the entire healthcare system (5). By the end of 2023, there were 5.6 million registered nurses in China, with only 4.0 registered nurses per 1,000 people (6). The “Healthy China 2030” planning outline proposes that by 2030, China will need to have 4.7 registered nurses per 1,000 people, which means there will be a shortage of at least 2 million nurses (7). In areas where there is a shortage of nurses, the infection rate of infectious diseases and the mortality rate of patients increase (8). The inadequate supply of nurses has notably created many negative impacts on the patient's health–related outcome as well as challenges to fight diseases and improving health, which results in decreasing the quality of nursing care, threatening the safety of patient and increasing the patient's mortality rate (9). Given the critical role of nurses in ensuring patient safety, understanding the factors that drive nurse turnover has become an urgent priority for healthcare systems worldwide. Among these factors, the quality of the work environment, particularly the safety climate, has emerged as a key determinant of nurse retention.

Turnover intention refers to the tendency of employees to leave their current job positions and seek other job opportunities (10); it is the best and most reliable antecedent variable for predicting turnover behavior (11). Previous studies have found that clinical nurses tend to have a high turnover intention (12). According to a cross-sectional study of 10 European countries, the turnover intention of nurses in these countries varies from 5 to 17% (13). A recent study by Al Sabei showed that 34% of nurses out of 2,113 indicated a desire to leave their jobs (14). A survey of clinical nurses in tertiary hospitals in China found that 48.29% of nurses had a high intention (15). Frequent nurse departures disrupt workforce stability, increase training costs, reduce organizational efficiency and care quality, and hinder the development of the nursing profession (16). Therefore, how to reduce nurses' turnover intention to alleviate the dilemma of nurse staffing shortage, and thus ensure the quality and sustainability of healthcare services, has become an important issue that needs to be prioritized in China and even globally.

Hospital safety climate refers to the overall perception of hospital staff regarding the safety of the work environment (17). The work environment serves as the primary medium through which nurses perceive the hospital safety climate. It affects the quality of care and wellbeing of patients (18). Research has shown that a positive organizational safety climate can provide nurses with adequate support when safety incidents occur, alleviate anxiety and guilt, promote nurses' adaptation to work, reduce occupational stress, and increase nursing staff's job satisfaction (19). The hospital safety climate is the most important component of the hospital's organizational culture. Nurses' positive perception of the safety climate in their working environment can significantly enhance their sense of organizational support and health belonging (20). At the same time, a positive hospital safety climate can inspire nurses' professional identity, improve job satisfaction, and enable nurses to remain in their positions (21). Therefore, improving working conditions in hospitals and creating a positive safety climate are critical to maintaining adequate staffing levels, ensuring high-quality patient care, and reducing nurse turnover intention.

Work engagement is a positive, substantial, work-related mental state that includes vigor, dedication, and absorption (22). Individuals with high work engagement fully invest their physical, cognitive, and emotional resources into their work roles. Research suggests that a high level of work engagement can enhance job performance, job satisfaction, and emotional health and reduce turnover intention (23). In healthcare settings, work engagement has an important influence on the quality of care (24). Research has found that nurses‘ work engagement is positively correlated with high-quality care and patient safety (25). Enhancing nurses' work engagement not only promotes their physical and mental health, increases job satisfaction and work efficiency, ensures high-quality nursing services, but also effectively reduces turnover rates, thereby maintaining the stability of the nursing workforce (26).

Based on the relationship between hospital safety climate, work engagement, and turnover intention, this study employs the Job Demands-Resources (JD-R) model as a key theoretical framework to elucidate how hospital safety climate, work engagement, and nurses' turnover intention interact. The model is predicated on the premise that all characteristics of the work environment are to be divided into two distinct categories: job demands and job resources (27). Job demands are defined as the negative factors that drain an individual's energy at work, including work overload, role conflict, time pressure, etc. Job resources are the elements that help individuals achieve their work goals, support their personal development, and reduce job stress. Job resources realize their potential through motivational processes and foster high work engagement, exhibiting a positive correlation with work engagement (28). According to the gain path of the JD-R model, adequate job resources play a motivational role, stimulate work engagement and foster positive organizational outcomes (i.e., reduced turnover intention) (29). Hospital safety climate is regarded as a vital job resource, providing nurses with security, organizational support, and psychological safety. When nurses feel supported and have access to job resources, they are likely to develop intrinsic motivation, which fosters greater work engagement (30), then enhances their commitment to their work and ultimately reduces their turnover intention.

While the mediating role of work engagement between safety climate and turnover intention has been validated among coal miners (31), this line of research has obvious limitations in terms of research objects and scenario applicability. Specifically, the subjects of the aforementioned study are limited to coal mine employees, whose work scenarios and occupational characteristics differ significantly from those of nurses. As a special caring profession, nurses are faced with unique occupational attributes such as high-intensity workloads, complex nurse-patient relationships, and high emotional labor demands. These characteristics suggest that the safety climate perceived by nurses is no longer limited to traditional organizational factors, but more comprehensively covers multiple aspects, including the clinical work environment, patient-related factors, and organizational support systems. From the perspective of the Job Demands-Resources (JD-R) model, safety climate acts as a critical job resource that shapes employees' motivational processes. In contrast to physically hazardous occupations such as coal mining, where safety climate mainly functions to reduce physical risks and ensure occupational safety, nursing relies on safety climate to alleviate emotional exhaustion, improve professional support, and sustain work motivation. Consequently, the underlying mechanisms by which safety climate influences turnover intention through work engagement may differ substantially between nurses and coal miners, and empirical evidence within the nursing context remains insufficient to verify this chain pathway. Therefore, examining this mediating model among clinical nurses can not only fill the contextual gap but also further extend the applicability of the JD-R model in the nursing profession.

**Figure 1:**
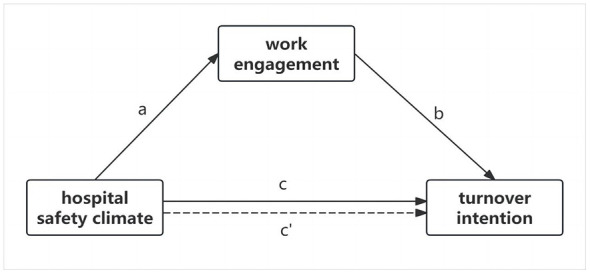
The theoretical model.

To address this research gap, the specific objectives of this survey were to (1) investigate the status of nurse turnover intention and (2) investigate the role of work engagement as a mediator in the relationship between the hospital safety climate and turnover intention among nurses in Guangdong Province. Guided by the Job Demands-Resources (JD-R) model and previous studies, we proposed a conceptual model diagram for this study, as shown in Figure 1. We hypothesized that (1) turnover intention is negatively correlated with hospital safety climate and work engagement and (2) work engagement plays a mediating role in the relationship between hospital safety climate and the turnover intention of nurses.

## 2 Methods

### 2.1 Study design and participants

From April to June 2025, a convenience sampling method was used for selecting nurses in Guangdong Province as the research objects. The following requirements have to be fulfilled by participants: (a) nurse with a license and (b) at least 1 year work experience in the hospitals. Nurses who were unwilling to participate in the study or suffering from mental illness were excluded. According to the Kendall sample estimation method, the sample size was 5–10 times the number of variables (32). A total of 28 variables (17 sociodemographic variables, 5 hospital safety climate variables, 3 work engagement variables, and 3 turnover intention variables) were examined in this study. A follow-up loss rate of 20% and a sample size of 168–336 were assumed. There were 8,600 questionnaires distributed in all, and 8,236 (95.77%) of them were properly returned.

### 2.2 Measures

The sociodemographic characteristics of the sample in the first part contained 17 variables, including Gender, Age, Marital status, Parenting situation, Educational level, Type of hospital, Hospital level, Hospital area, Department, Professional Title, Work experience, Employment mode, Monthly income, Specialist nurses, Monthly night shifts, Weekly working hours and Frequency of workplace violence.

The Hospital Safety Climate Scale (HSCS) was developed by Gershon (17) and translated and revised by the Chinese scholars Xu Na (33). It has 21 items measuring 5 dimensions, including management support, absence of job hindrances, feedback and training, cleanliness and orderliness, minimal conflict and good communication. Likert 5-level scoring method is adopted, with 1 indicating “strongly disagree” to 5 indicating “strongly agree”. Higher hospital safety climate is indicated by a higher score. The scale's Cronbach's alpha in this research was 0.972.

The Work Engagement Scale (WES) was developed by Schaufeli (34) and its validity and reliability of Chinese version were confirmed by Zhang Yiwe (35), contains 15 items in 3 dimensions: vigor, dedication and absorption. The scale uses a Likert 7-point rating scale, with scores ranging from 0 to 6 indicating “never”, “almost never”, “rarely”, “sometimes”, “often”, “very often” and “always”. Higher work engagement is indicated by a higher score. The scale's Cronbach's alpha in this research was 0.967.

The Turnover Intention Scale (TIS) was developed by Michaels and Spector (36) and translated and revised by the Taiwanese scholars Li Dongrong and Li Jingyuan (37). The scale consists of 6 items, where items 1 and 6 form turnover intention I dimension, investigating the possibility of withdrawing from the current job; items 2 and 3 form turnover intention II dimension, investigating the motivation to find another job; items 4 and 5 form turnover intention III dimension, investigating the possibility of getting an external job. The scale uses a Likert 4-point rating scale with scores ranging from 1 to 4 indicating “never”, “rarely”, “occasionally” and “often”. Higher score indicates a stronger willingness to leave. The mean item score of intention to leave was classified according to the criteria proposed by Tang (38) in 2022: very low ( ≤ 1), low (>1 to ≤ 2), high (>2 to ≤ 3), and very high (>3). This classification has been used in previous studies on turnover intention among nursing populations. The scale's Cronbach's was 0.909.

### 2.3 Data collection

A web-based questionnaire platform called “Survey star” was used to conduct the survey. It was designed for avoiding respondents from being questioned beyond once. An email inviting administrators at hospitals to take part in the research was sent to them. The purpose of the study was explained to participants, and they were assured that the information they provided would remain confidential. The questionnaires could only be completed once the per internet protocol (IP) address was identified and only questionnaires with all questions completed could be submitted.

### 2.4 Data analysis

Data analysis was performed using IBM SPSS 27.0 and AMOS 26.0.

1. The continuous data were described by the mean (M) and standard deviation (SD) if they were normally distributed. If the continuous data were not normally distributed, then they were described by the median (Mdn) and interquartile range (IQR). Categorical variables were described using frequency and percentage.
2. Common method bias test: Since all core variables were collected through self-reported questionnaires at a single time point, there was a potential risk of common method bias. Harman's single-factor test was adopted for evaluation; all items of all research scales were summarized and subjected to unrotated exploratory factor analysis, and the severity of bias was judged by the variance explanation rate of the first common factor.
3. Confirmatory factor analysis was conducted for all core variables (hospital safety climate scale, work engagement scale, turnover intention scale) respectively. The test standards referred to general academic standards (RMSEA < 0.08, CFI>0.90, TLI>0.90) to verify the construct validity of the scales.
4. Pearson correlation analysis was applied to examine the correlation among hospital safety climate, work engagement and turnover intention.
5. Finally, we used PROCESS V.3.5 Macro in SPSS (Model 4) to test the mediation model and the direct and indirect effects of the hospital safety climate on turnover intention were examined using bootstrap analyses with 5,000 bootstrap samples and 95% confidence intervals.

It should be noted that this study included more than 8,000 valid samples, which is a large-sample study. In the preliminary analysis, we attempted to construct a structural equation model (SEM) to simultaneously test the measurement validity and the structural model. However, due to the distribution characteristics of large-sample data and model settings, the model fit indices did not meet the ideal standards, resulting in poor fitting effect. Compared with SEM, the PROCESS macro has better stability and robustness in exploratory mediation analysis with large samples, and its estimation of effect values is concise and reliable. Therefore, this study ultimately adopted this method for mediation effect testing. Meanwhile, limited by the analytical strategy, this study did not simultaneously conduct an overall verification of the measurement model and the structural model, and the research method is relatively simplified. In addition, this study did not include potential important confounding variables such as age, workload, workplace violence, and hospital level in the model. The above factors may interfere with the relationships between variables, leading to certain bias in the estimation of mediation effects, which should be considered when interpreting the results.

## 3 Results

### 3.1 Demographic characteristics

A total of 8,600 nurses participated and answered the questionnaire; 8,236 were valid, with an effective rate of 95.77%. The data showed that 341 (4.1%) males and 7,895 females (95.9%) participated in the study. In terms of education, 258 (3.1%) possessed a technical secondary school degree, 2,822 (34.3%) possessed a junior college degree, 5,122 (62.2%) possessed a bachelor's degree, 34 (0.4%) possessed a master's degree and above. Table 1 displays the remaining demographic features of this sample.

**Table 1:** General characteristics of the nurse (*N* = 8236).

Variables	Categories	Frequency (*n*)	Percentage (%)
Gender	Male	341	4.1
	Female	7895	95.9
Age	18–25	1305	15.8
	26–30	2326	28.2
	31–35	2062	25.0
	36–40	1021	12.4
	≥41	1522	18.5
Marital status	Single	2631	31.9
	Married	5443	66.1
	Divorced/separated	162	2.0
Parenting situation	Childless	3113	37.8
	One child	2298	27.9
	Two children and above	2825	34.3
Educational level	Technical secondary school	258	3.1
	Junior colllege	2822	34.3
	Bachelor's degree	5122	62.2
	Master's degree and above	34	0.4
Type of hospital	General hospital	7279	88.4
	Specialized hospital	957	11.6
Hospital level	Primary hospital	1168	14.2
	Secondary hospital	2982	36.2
	Tertiary hospital	4086	49.6
Hospital area	The pearl river delta	2980	36.2
	Eastern Guangdong	1433	17.4
	Western Guangdong	1837	22.3
	Northern Guangdong	1986	24.1
Department	Internal Medicine	2236	27.1
	Surgery	1475	17.9
	Obstetrics and Gynecology	705	8.6
	Pediatrics	457	5.5
	Emergency Department	658	8.0
	ICU	503	6.1
	Operating room	378	4.6
	Others	1824	22.1
Professional Title	Nurse	1966	23.9
	Primary nurse	3179	38.6
	Nurse-in-charge	2559	31.1
	Associate Chief Nurse	455	5.5
	Chief Nurse	77	0.9
Work experience (in years)	1–2	955	11.6
	3–5	1212	14.7
	6–10	2137	25.9
	11–20	2680	32.5
	≥21	1252	15.2
Employment mode	Temporary Agency Work	246	3.0
	Contract system	5614	68.2
	Tenure-track	2376	28.8
Monthly income (yuan)	< 6000	3400	41.3
	6001–9000	3246	39.4
	9001–12000	1107	13.4
	12001–15000	323	3.9
	≥15001	160	1.9
Specialist nurses	Yes	999	12.1
	No	7237	87.9
Monthly night shifts (times per month)	0	1492	18.1
	1–4	3161	38.4
	5–7	2511	30.5
	≥8	1072	13.0
Weekly working hours (hours per week)	< 40	1497	18.2
	41–45	3928	47.7
	46–50	1839	22.3
	≥51	972	11.8
Frequency of workplace violence (times per year)	0	5670	68.8
	1–3	1924	23.4
	4–6	262	3.2
	≥7	380	4.6

### 3.2 Results of common method bias test

The results of Harman's single-factor test showed that the variance explanation rate of the first common factor was 47.267%. Although this value is slightly higher than the empirical threshold of 40%, suggesting a certain degree of common method bias, it does not exceed the critical value of 50%, indicating that there is no serious common method bias in this study, and its interference on the research results is within an acceptable range, so no additional bias control measures are needed. See Table 2 for details.

**Table 2:** Results of common method bias test.

Component	Initial eigenvalues			Extraction sums of squared loadings		
	Total	% of variance	Cumulative %	Total	% of variance	Cumulative %
1	19.852	47.267	47.267	19.852	47.267	47.267
2	5.584	13.295	60.561	5.584	13.295	60.561
3	3.230	7.690	68.251	3.230	7.690	68.251
4	1.123	2.674	70.925	1.123	2.674	70.925

### 3.3 Results of confirmatory factor analysis

As shown in Table 3, all fitting indexes of the scales met the general academic standards, indicating that each scale has good construct validity and reliable measurement results. The specific fitting indexes are as follows: Hospital Safety Climate Scale (RMSEA = 0.078, CFI = 0.952, TLI = 0.944); Work Engagement Scale (RMSEA = 0.078, CFI = 0.975, TLI = 0.960); Turnover Intention Scale (RMSEA = 0.077, CFI = 0.991, TLI = 0.979).

**Table 3:** Results of confirmatory factor analysis.

Fit indices	Reference standards	Hospital safety climate	Work engagement	Turnover intention
RMSEA	≤0.08	0.078	0.078	0.077
SRMR	≤0.08	0.033	0.024	0.020
CFI	≥0.90	0.952	0.975	0.991
TLI	≥0.90	0.944	0.960	0.979

### 3.4 Scores of hospital safety climate, work engagement and turnover intention

The total score of hospital safety climate was 91.51 ± 13.01, with a mean item score of 4.36 ± 0.62, and the dimension score were 4.43 ± 0.64 for management support, 4.12 ± 0.85 for absence of job hindrances, 4.43 ± 0.62 for feedback and training, 4.29 ± 0.73 for cleanliness and orderliness, and 4.37 ± 0.69 for minimal conflict and good communication. The total score for work engagement was 68.26 ± 21.40, and the mean score across all items was 4.55 ± 1.43. The score for the vigor dimension was 4.76 ± 1.45, the score for the dedication dimension was 4.33 ± 1.57, and the score for the absorption dimension was 4.49±1.51. The total score for turnover intention was 12.54 ± 4.72, and the mean score across all items was 2.09 ± 0.79. Specifically, the score for the turnover intention I dimension was 1.97 ± 0.90, the score for the turnover intention II dimension was 2.02 ± 0.87, and the score for the turnover intention III dimension was 2.28 ± 0.88.

### 3.5 Correlation analysis of hospital safety climate, work engagement and turnover intention in nurses

As seen in Table 4, higher levels of turnover intention were associated with lower hospital safety climate (*r* = −0.324, *P* < 0.001) and lower work engagement (*r* = −0.400, *P* < 0.001). There was a positive correlation between hospital safety climate and work engagement (*r* = 0.549, *P* < 0.001).

**Table 4:** Pearson correlations among hospital safety climate, work engagement and turnover intention (*N* = 8236).

Variables and dimensions	1	2	3	4	5	6	7	8	9	10	11	12	13	14
1	–													
2	0.922^**^	–												
3	0.862^**^	0.729^**^	–											
4	0.945^**^	0.856^**^	0.740^**^	–										
5	0.885^**^	0.726^**^	0.768^**^	0.793^**^	–									
6	0.872^**^	0.731^**^	0.688^**^	0.802^**^	0.785^**^	–								
7	0.549^**^	0.474^**^	0.519^**^	0.486^**^	0.525^**^	0.501^**^	–							
8	0.552^**^	0.476^**^	0.524^**^	0.486^**^	0.532^**^	0.507^**^	0.946^**^	–						
9	0.501^**^	0.429^**^	0.481^**^	0.441^**^	0.483^**^	0.454^**^	0.957^**^	0.856^**^	–					
10	0.503^**^	0.440^**^	0.466^**^	0.452^**^	0.473^**^	0.458^**^	0.947^**^	0.816^**^	0.894^**^	–				
11	−0.324^**^	−0.281^**^	−0.331^**^	−0.274^**^	−0.303^**^	−0.296^**^	−0.400^**^	−0.372^**^	−0.375^**^	−0.391^**^	–			
12	−0.349^**^	−0.307^**^	−0.344^**^	−0.296^**^	−0.322^**^	−0.326^**^	−0.431^**^	−0.404^**^	−0.400^**^	−0.420^**^	0.915^**^	–		
13	−0.307^**^	−0.266^**^	−0.303^**^	−0.265^**^	−0.286^**^	−0.282^**^	−0.381^**^	−0.354^**^	−0.358^**^	−0.370^**^	0.916^**^	0.818^**^	–	
14	−0.211^**^	−0.179^**^	−0.237^**^	−0.170^**^	−0.203^**^	−0.184^**^	−0.258^**^	−0.236^**^	−0.244^**^	−0.256^**^	0.845^**^	0.628^**^	0.637^**^	–

1, hospital safety climate; 2, management support; 3, absence of job hindrances; 4, feedback/training; 5, cleanliness/orderliness; 6, Minimal conflict/good communication; 7, work engagement; 8, vigor; 9, dedication; 10, absorption;11, turnover intention; 12, turnover intention dimension I; 13, turnover intention dimension II; 14, turnover intention dimension III.

^**^p < 0.01.

### 3.6 Mediation analysis between hospital safety climate, work engagement and nurses' turnover intention

**Figure 2:**
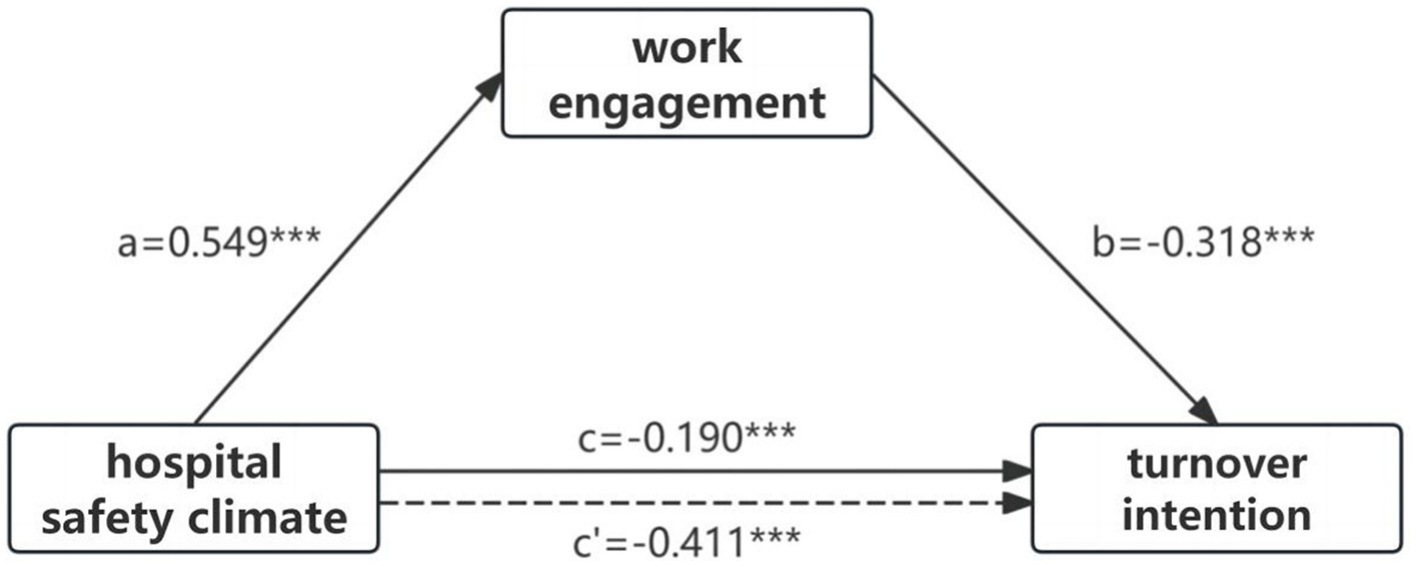
The mediating effect model of work engagement between hosiptal safety climate and turnover intention (*N* = 8236). ***p < 0.001.

The results demonstrated that the 95% CI for both the direct and indirect effects of hospital safety climate on nurses' turnover intention did not include zero, indicating that work engagement partially mediated the relationship between hospital safety climate and turnover intention. The direct effect of hospital safety climate on turnover intention was−0.190, the indirect effect was−0.175, the total effect was−0.411, and the percentage of mediation effect was 53.77% (see Table 5). The mediating effect model of work engagement between hospital safety climate and turnover intention is shown in Figure 2.

**Table 5:** The mediating effects of work engagement on hospital safety climate and turnover intention (*N* = 8236).

Effect	Path	B	SE	*P*	95%CI	Percent(%)
Direct effect	HSC-TI	−0.190	0.015	<0.001	−0.219 −0.160	46.23%
Indirect effect	HSC-WE-TI	−0.175	0.008	–	−0.190 −0.159	53.77%
Total effect		−0.411	0.013	< 0.001	−0.437 −0.385	

HSC, hospital safety climate; WE, work engagement; TI, turnover intention.

## 4 Discussion

The present study investigated the status of nurses' turnover intention in Guangdong Province and tested the mediating role of work engagement in the association between hospital safety climate and turnover intention based on the JD-R model. Consistent with the hypothesis, hospital safety climate and work engagement were significantly negatively associated with turnover intention, while hospital safety climate was positively related to work engagement. Work engagement significantly mediated the relationship between hospital safety climate and turnover intention. The results of this study may inform nurse managers to reduce nurses' intention to leave and develop nursing interventions from the perspective of hospital safety climate.

### 4.1 Current status of nurses' turnover intention

In this study, the mean item score of turnover intention among nurses was 2.09 ± 0.79. According to the classification criteria described in the Methods section, the turnover intention among nurses in Guangdong Province is at a high level. This score is similar to the results of a survey conduct by Zhou on nurses' turnover intention in tertiary Grade A hospitals in Sichuan Province, China (39). The elevated turnover intention is associated with several factors. As a province with a large population inflow, hospitals in Guangdong Province experience high patient volumes year-round, resulting in high work intensity and many night shifts for nurses, which aggravate their workload. In addition, the ongoing reform of the remuneration system is associated with reduced nurses' professional identity, which is further positively correlated with the formation of turnover intention. Previous research has showen that job stress and professional identity are important predictors of nurses' turnover intention (40). Among the mean scores of the dimensions, Turnover Intention III (2.28 ± 0.88) scored the highest, and Turnover Intention I (1.97 ± 0.90) scored the lowest. This indicates that nurses in Guangdong have relatively high external employment possibilities but are relatively reluctant to leave their current positions. Combined with the regional characteristics of Guangdong's medical and social environment, the reasons for this are potentially associated with the following aspects: first, Guangdong, as a major economic and medical hub in southern China, has a rapid development of medical and health undertakings, a large number of private hospitals, specialized clinics and older adults care institutions, which is correlated with the further expansion of employment space for nurses and linked to enhanced external employment advantages. Second, nursing is more universality than other professions in terms of specialized skills and vocational qualifications, which is associated with nurses having more choices in the job market. At the same time, factors such as the relatively job stability of public hospitals in Guangdong and the high material and living needs in the Pearl River Delta region are potentially ralated to their hesitation to actively pursue alternative employment.

### 4.2 Correlation among hospital safety climate, work engagement and nurses' turnover intention

Our analysis indicates that hospital safety climate was positively correlated with work engagement; i.e., the greater the degree of hospital safety climate, the higher the work engagement was. Consistent with previous studies (41). As the primary providers of medical services, nurses are frequently exposed to infectious environments, and the perceived safety climate within hospitals is closely associated with their safety-related behaviors (42). Guangdong has a large number of tertiary hospitals and specialized medical institutions, with a high patient flow and a heavy clinical workload for nurses, making their perception of safety climate more closely related to their work state. Many hospitals in Guangdong are actively promoting medical quality and safety improvement projects in line with the provincial nursing development plan, and the improvement of safety management systems, adequate allocation of safety protection equipment, and smooth safety feedback channels are associated with the alleviation of physical and psychological pressure of nurses working in high-intensity environments, which in turn enhances their work engagement. Improving hospital safety climate, for instance by implementing better safety management systems, providing adequate safety protection equipment, and establishing smooth safety feedback channels, is associated with a reduction in the physical and psychological stress on nurses. This is related to nurses feeling more secure and have a greater sense of belonging in their work, which is further associated with increased commitment to work and the provision of higher quality nursing services to patients. This study also confirmed that work engagement was negatively associated with turnover intention. Nurse engagement at work is a closely associated with hospital nurse intent to leave (43) and is positively correlated with the quality of nursing service (44). In Guangdong, the negative association between work engagement and turnover intention is closely related to the regional labor market characteristics. As an economically developed region, Guangdong has a strong demand for nursing talents, and nurses have more job options in the local medical market. When nurses lack work engagement and sense of belonging, they are more likely to switch jobs to seek better development opportunities, which further highlights the role of work engagement in reducing turnover intention among Guangdong nurses. In addition, Guangdong's aging population is accelerating, and the demand for older adults care and specialized nursing services is increasing, which puts higher requirements on nurses' work engagement; nurses with low engagement are more likely to have turnover intentions due to the inability to adapt to the increasing work demands. Employees who are highly dedicated are characterized as having a strong sense of pride, purpose, and inspiration, and it is an indicator of job satisfaction (45). Related studies have shown that nurses' job satisfaction, as an attitudinal response to their work, plays a key role in their retention intention (46).

### 4.3 The mediating effect of work engagement on the relationship between hospital safety climate and turnover intention among nurses

The results confirmed that work engagement plays a partial mediating role between hospital safety climate and turnover intention. Hospital safety climate is negatively correlated with turnover intention both directly and indirectly, with the indirect association linked to higher work engagement. As a core component of hospital organizational culture, when nurses perceive an adverse safety climate, they are prone to experiencing occupational burnout and psychological stress in a work environment lacking effective safety guarantees, which is associated with lower levels of work engagement, and this is further correlated with high turnover intention. According to the Job Demands-Resources (JD-R) model, hospital safety climate can be regarded as a key job resource, which is associated with sufficient psychological safety for nurses, correlated with the alleviation of work insecurity and chronic stress, and further positively associated with nurses' initiative and enthusiasm for work engagement. As a positive psychological resource, work engagement is a core indicator for measuring organizational health and employee wellbeing (47). A high level of work engagement can enhance nurses' sense of identity and satisfaction with their work, enabling them to gain a sense of accomplishment and value in nursing practice (48). When nurses perceive a positive hospital safety climate, they will truly feel the hospital's attention and care for their occupational safety (49). This psychological perception of being valued will be transformed into continuous work motivation, which is correlated with nurses' work engagement (50), linked to their earnest performance of job responsibilities, and thereby enhance their willingness to stay in the position.

Although the R^2^ values in the present model were relatively low, this is common and acceptable in cross-sectional studies related to psychology and occupational behavior. Turnover intention is a complex outcome affected by individual characteristics, family conditions, salary, career development, organizational policy, and other unmeasured variables. In addition, this study did not include potential confounding variables such as age, workload, workplace violence, and hospital level in the model; these factors may interfere with the associations among core variables, which may further lead to certain bias in the estimation of mediation effects and is one of the potential reasons for the relatively low R^2^ value of the model. The present study focused on the specific mediating mechanism of job resources and psychological energy rather than explaining all variance of turnover intention. Consistent with our hypotheses, the direct effect of hospital safety climate on turnover intention (B = −0.190) and the indirect effect via work engagement (B = −0.175, accounting for 53.77% of the total effect) were both statistically significant, and the mediating pathway was stable and meaningful. Therefore, the relatively low R^2^ does not affect the validity of the model or the reliability of the findings. Instead, it reflects the multi-factorial nature of nurses' turnover intention and suggests that further research incorporating more predictors is warranted.

This study aimed to reduce nurses' turnover intention by improving their work engagement through enhancing the hospital safety climate. To achieve this goal, the health care system and nursing managers must work together to strengthen the investment in nursing and improve nursing work environments and the career development system, so as to ensure a stable and healthy nursing workforce. Hospitals should pay attention to the occupational safety of nurses, by establishing a well-developed management system and a proactive safety communication environment to create a positive hospital safety climate. In this way, nurses' motivation and initiative can be improved, which will encourage them to devote more energy to their work and ultimately increase the retention rate.

### 4.4 Limitations

Our study had several limitations. First, the study was conducted exclusively among nurses in hospitals in Guangdong Province, China. Guangdong has a relatively developed medical and health system. This means the working environment, safety management mechanisms, and welfare benefits for nurses here may differ significantly from those in central and western regions of China. In addition, the sample selection may be biased toward tertiary hospitals and general hospitals, with insufficient coverage of primary medical institutions and specialized hospital, which limits the external validity of the conclusions and prevents direct generalization of the findings to nurses in other provinces, regions, or different levels of hospitals in China. Second, this study employed a cross-sectional design. As all variables were measured at a single time point, the temporal relationship among hospital safety climate, work engagement and turnover intention could not be established, thereby precluding any causal inferences. Third, although quality control was in place prior to the distribution of the questionnaires, it is possible that some nurses might not have been fully candid when answering the online questionnaires, potentially introducing response bias. Moreover, all variables were collected via self-reported questionnaires in a single survey, creating a potential risk of common method bias, even though this study supplemented Harman's single-factor test to evaluate this bias, residual bias may still exist; future studies can adopt multi-source data collection to further reduce the impact of common method bias. Fourth, this study used Hayes' PROCESS macro for mediation analysis, which is relatively simple in terms of methodology. Although the results of this method are more robust under large samples, due to the poor fitting effect of the structural equation model in the preliminary stage, it was not possible to simultaneously test the measurement model and the structural model, leaving room for improvement in methodological rigor. Future studies can improve model fitting by optimizing the scale structure and adjusting model settings, and adopt structural equation modeling (SEM) for more comprehensive verification analysis. Fifth, this study did not strictly control confounding factors such as age, workload, workplace violence, and hospital level. The above variables may affect the correlation strength between core variables, and future studies can control them in multivariate models to improve the reliability of the results.

## 5 Conclusion

In summary, hospital safety climate and work engagement are negatively related to turnover intention. Work engagement mediates the relationship between hospital safety climate and turnover intention. The results of this study suggest that nurse managers should pay attention to the core needs of nurses, formulate relevant policies, improve the safety management system, create a positive hospital safety climate, effectively guarantee the occupational safety of nurses, which will help enhance work engagement, thereby reducing turnover intention, stabilizing the nursing team, and promoting the healthy and sustainable development of the nursing profession.

## Data Availability

The original contributions presented in the study are included in the article/supplementary material, further inquiries can be directed to the corresponding authors.
